# Treatment of progressive multifocal leukoencephalopathy

**DOI:** 10.1007/s00415-019-09501-y

**Published:** 2019-08-17

**Authors:** Daniel Castle, Neil P. Robertson

**Affiliations:** Institute of Psychological Medicine and Clinical Neuroscience, Cardiff University, University Hospital of Wales, Heath Park, Cardiff, C14 4XN UK

Progressive multifocal leukoencephalopathy (PML) is a rapidly progressive demyelinating disease of the central nervous system caused by John Cunningham polyomavirus (JCV), a globally seroprevelant virus that commonly causes a silent or benign infection. However, in immunocompromised patients JCV can be reactivated. In this situation, most organs remain unaffected, but multiplication within oligodendrocytes causes lytic brain lesions resulting in progressive motor dysfunction, cognitive impairment, and visual deficits. Although some strategies are available for treatment, particularly if recognised early, PML has an overall mortality of 30–50% and can result in substantial morbidity.

Progressive multifocal leukoencephalopathy is thankfully rare and most commonly associated with lymphoproliferative disease or advanced human immunodeficiency virus (HIV) infection. However, PML is now well known to be associated with the use of disease modifying therapies (DMTs) in multiple sclerosis (MS) such as natalizumab that alter cell-mediated immunity. Given the serious implications of a diagnosis of PML, patients now undergo a process of risk stratification at commencement of treatment and then selective serological testing over the course of treatment, interval magnetic resonance imaging (MRI) and regular clinical surveillance to identify any early pathological changes. Once a diagnosis is established, current treatment strategies include withdrawal of immunosuppressive therapies, plasma exchange (PLEX), mirtazapine, mefloquine as well as strategies to up regulate patient immunity.

This month’s journal club reviews three new potential treatment options in the management of PML. The first paper reviews the use of pembrolizumab, the second allogeneic BK virus-specific T cells and the third filgrastim.

## Pembrolizumab treatment for progressive multifocal leukoencephalopathy

Pembrolizumab is a monoclonal antibody used as an immunotherapy in conditions such as melanoma or non-small cell lung cancer. It is designed to block the expression of programme cell death protein 1 (PD-1) expressed on the surface of T cells. This protein acts as a negative regulator of immune cells and prolonged expression, such as that observed in chronic infections can lead to suppression of T cell activity and repertoire. Significantly, PD-1 expression was found to be up-regulated on CD4+ and CD8+ cells in patients with PML and is specifically enriched on JCV-specific CD8+ cells. It was therefore hypothesised that blockade of PD-1 in PML would allow reinvigoration of the T cell response and suppression of PML.

Eight adults with PML were identified and had a range of underlying conditions including chronic lymphoid leukaemia, advanced HIV infection, Hodgkin’s and non-Hodgkin’s lymphoma and idiopathic lymphopenia. The majority had been treated with chemotherapy or DMT agents in the past. All continued to have detectable viral loads of JCV in the cerebral spinal fluid (CSF) ranging from 63 to 28,350 copies per millilitre at entry into the study. Pembrolizumab was given intravenously at a dose of 2 mg per kg every 4–6 weeks, for a maximum of three doses. Clinical evaluations included neurologic examination, use of a modified Rankin scale to assess disability, MRI, CSF and blood testing.

Five of the eight patients exhibited clinical improvement or stabilisation of neurological symptoms, reduction in CSF JCV viral load and corresponding stabilisation or reduction in lesion burden on MRI brain. One exhibited no clinical improvement, but in retrospect was noted to have had some clinical, radiologic, and virologic stabilisation of PML before treatment. The remaining two patients worsened clinically and subsequently died. No patients had complete resolution of PML brain lesions. Apart from two patients who developed a rash, the drug was well tolerated.

*Comment*: This novel study repurposed an existing cancer immunotherapy following important pathophysiological observations by the authors. This study is clearly limited by its sample size and heterogeneous patient cohort but offers some promise as well as a useful model for evaluation of novel treatments in PML. Although difficult, given the rarity of PML, future studies exploring more specified patient groups, such as Natalizumab induced PML in MS as well as development of a standardised way of determining and monitoring disease state and severity would be of value.

Cortese I et al (2019) N Engl J Med 380:1597–1605.

## Allogeneic BK virus-specific T cells for progressive multifocal leukoencephalopathy

The BK virus belongs to the same polyomaviridae family as JCV and is genetically similar with a number of shared immunogenic proteins. Clinically BK virus causes nephritis and cystitis in patients who have undergone solid organ or stem-cell transplantation. BK virus has previously been treated with viral-specific T cells and it was hypothesised that given the shared epitopes of BK and JCV that this treatment could also be successful in PML.

This study utilised BK virus-specific T cells generated from 27 healthy donors. The most closely HLA-matched T cell line for each patient was chosen and via intravenous infusion, a dose of 2 × 10^5^ T cells per kg every 4 weeks was given until JCV was cleared from the CSF. Three patients with PML were identified for the study, the first had a diagnosis of acute myeloid leukaemia treated with cord–blood transplant the second patient had a myeloproliferative neoplasm treated with ruxolitinib and the third patient had chronic HIV infection previously treated with highly active antiretroviral therapy.

After treatment, two patients had an improvement in clinical status, imaging features of PML and in both JCV was successfully cleared from the CSF. Both patients also had radiological evidence of immune reconstitution inflammatory syndrome (IRIS), but no relevant clinical manifestations. The third patient had a reduction in JC viral load and symptom stabilisation but died 8 months after the last infusion; cause of death was not reported.

*Comment*: This proof of concept study confirmed that JCV can be cleared from CSF and the clinical features of PML improved using a third party partial HLA matched T cell specific BK virus treatment. These positive responses occurred despite evidence of persistent T cell immunodeficiency, even with radiological evidence of IRIS suggesting inflammatory upregulation at the site of PML infection caused by the infused T cells. Supportive evidence was also offered by measurement of the infused T cells in the CSF for more than 250 days after treatment. No graft versus host disease was observed although this may have been as the result of the immunocompromised state of the patients. Although patient specific T cell treatments may improve outcomes and reduce the risk of inflammatory sequelae the use of ‘off-the-shelf’ medication as in this study, provides a novel rapid method to treat this debilitating condition.

Muftuoglu M et al (2018) N Engl J Med 379:1443–1451.

## Treatment of natalizumab‐associated PML with filgrastim

As of December 2018, there have been 801 confirmed cases of natalizumab induced PML in MS, with mortality rates of 8–29%. Filgrastim (also known as granulocyte-colony stimulating factor) is used widely to upregulate the immune system after chemotherapy. It promotes production of granulocytes, lymphocytes and antigen presenting cells, while also increasing adhesive properties of T cells independent of integrin α4β1; the adhesin blocked by natalizumab.

This study was a retrospective analysis of 17 natalizumab induced PML cases in MS from 2010 to 2017 in a single centre. PML occurred on average after 49 infusions of natalizumab. Fifteen patients had clinical features consistent with PML and two were identified through routine surveillance MRI. Following diagnosis natalizumab was stopped in all patients, 8 underwent PLEX, 14 received mefloquine and 15 received Mirtazapine. All patients were then treated with daily filgrastim 5 µg/kg subcutaneously until baseline absolute lymphocyte counts were approximately doubled, which occurred at a mean of 9.94 days. Once PML-IRIS was confirmed (at a mean of 57.4 days) in 15 patients, they were also treated with intravenous Methylprednisolone followed by a tapering dose of oral corticosteroids. Nine of these patients were also treated with maraviroc to dampen the IRIS response. Using a Karnofsky functionality scoring system, seven patients recovered to or near the PML diagnosis baseline, three improved, but not to baseline functionality and seven had poor outcomes requiring full care. There was no evidence for the worsening of MS with any of the above procedures, with three patients having an MS relapse within a year of PML diagnosis.

*Comment*: This retrospective study explored the open label use of filgrastim in natalizumab induced PML. Although 100% of patients survived for at least 2 years, the use of a variety of parallel treatment regimes limits interpretation of outcomes and the significance of any additional beneficial effect for filgrastim in these circumstances. Nevertheless, careful surveillance for PML, immune activation by filgrastim and subsequent PML-IRIS management in combination appeared beneficial. Further work to define the most appropriate management strategy would be of value. In addition, the use of a relatively homogenous MS cohort was useful in order to provide clinicians with informed strategies to manage natalizumab induced PML and council patients as to the risk of their MS deteriorating.

Stefoski D et al (2019) Ann Clin Transl Neurol 6: 923–931.

